# Dr. Thomas Francis Jr.: A Legacy of Vaccine Innovation and Public Health Advancement

**DOI:** 10.7759/cureus.74451

**Published:** 2024-11-25

**Authors:** Priyanka Jali, Manjusha S Deshpande, Priyanka Khopkar-Kale, Srikanth Tripathy

**Affiliations:** 1 Central Research Facility, Dr. D. Y. Patil Medical College, Hospital and Research Centre, Dr. D. Y. Patil Vidyapeeth (Deemed to Be University), Pune, IND

**Keywords:** historical vignette, influenza vaccine, polio vaccine, preventive actions, public health and safety

## Abstract

Dr. Thomas Francis Jr. was an American physician, virologist, and epidemiologist who was a professor of epidemiology at the University of Michigan from 1941 to 1969. He focused on studying infectious diseases, particularly pneumonia, influenza, and poliomyelitis, and developing vaccines and clinical trials. He earned his medical degree from Yale University in 1925. In 1941, the Armed Forces Epidemiological Board (AFEB) appointed him as the Director of the Commission on Influenza. This prestigious role underscored his expertise and leadership in combating infectious diseases and successfully developing field trials and evaluations of protective influenza vaccines. In 1954, he spearheaded the largest and most comprehensive clinical trial of the Salk polio vaccine at the University of Michigan Vaccine Evaluation Center, with vital support from the National Foundation for Infantile Paralysis (NFIP). He received the Medal of Freedom in 1946, the Lasker Award in 1947, and the Medal in Global Public Health in 2005.

## Introduction and background

Dr. Thomas Francis Jr. was an American physician, virologist, and epidemiologist born on July 15, 1900 [1]. He was a professor of epidemiology at the University of Michigan from 1941 to 1969, and he developed influenza and poliomyelitis vaccines. He was a great student who completed his education with a scholarship from Allegheny College in Meadville, Pennsylvania (United States of America) and Yale Medical School in New Haven, New York (USA). His work focused on studying the causes of infectious diseases, especially pneumonia, influenza, and poliomyelitis, and developing vaccines and clinical trials for these diseases [2]. The aim of this article is to highlight the significant contributions to the global health community, medical science, public health, and the achievements of the research field.

## Review

Early life and career

Thomas was born in Gas City, Indiana, United States of America, on July 15, 1900, the son of Thomas Francis Sr. and Elizabeth (Cadogan) Francis. He was the third of four children in his family. His father, Thomas Francis Sr., began his studies to enter the ministry. However, he later decided to follow a different path and joined his father in working in the tin mills of South Wales. His father was a steelworker who also devoted time to serving as a part-time minister. His mother, Elizabeth Francis, had completed her graduation from the Salvation Army Training School in London. Francis thrived in western Pennsylvania, graduating from New Castle High School in New Castle, Indiana (USA), in 1917. He pursued higher education at Allegheny College (USA), earning a scholarship and graduating in 1921. He then obtained his medical degree from Yale University (New York) in 1925 [3]. In college, he competed as an amateur boxer. He was an astute, attractive, lively, and rapid learner pupil. He also recognized that the nation and Yale University wanted the Yale School to spearhead a reform movement in medical education. At Yale University School of Medicine, he worked with Dr. Blake, beginning his clinical and academic journey with a two-year residency at New Haven Hospital under the mentorship of Francis G. Blake, who chaired Yale's Department of Internal Medicine. His inspiration stemmed from Dr. Blake, the inaugural president of the Army Epidemiological Board (AEB) during World War II and subsequent years. Dr. Blake's early recognition of Francis's exceptional talent proved prescient; sixteen years later, Francis ascended to the presidency of the American Society for Clinical Investigation. This trajectory not only attests to his remarkable capabilities but also underscores the profound impact of mentorship in shaping the careers of emerging scientists. Impressed by Francis's promise, Blake hired him as an instructor in 1927. Francis took on a new challenge as an assistant at the Rockefeller Institute Hospital (New York, USA), where he worked under Rufus Cole and O.T. Avery in the Clinical Pneumonia Department in 1928. By 1931, his exceptional contributions earned him the rank of associate. Francis married Dorothy Packard Otton in 1933, and they had two children, Mary Jane and Thomas Francis III ("T") [2]. In 1936, he joined the International Health Division of the Rockefeller Foundation (IHDRF) in New York City (USA), set up a study laboratory, and began researching the etiology and epidemiology of influenza [4]. He was chief of the Department of Bacteriology at New York University College of Medicine and a professor there from 1938 to 1941. He took on the crucial position of Director of the Armed Forces Epidemiological Board's (AFEB) Commission on Influenza in 1941. He was able to significantly contribute to creating, testing, and assessing protective influenza vaccinations in this capacity. Henry F. Vaughan invited him to join the University of Michigan's recently constructed School of Public Health the same year. There, he established an Epidemiology Department and a Virus Laboratory devoted to researching numerous infectious diseases [2]. In 1947, he was honored with a position as Henry Sewell Professor of Epidemiology. He was the AFEB's president from 1958 to 1960 (AFEB) [4]. He continued his work at the University of Michigan until his death on October 1, 1969 [3].

Contribution of Thomas Francis to Pneumonia Vaccine Development

Thomas Francis Jr. revolutionized the fields of microbiology and epidemiology, making groundbreaking contributions to our understanding of bacterial infections, especially those caused by Streptococcus pneumoniae. His pioneering research laid the foundation for critical advances in preventing and treating these deadly infections. He continued his research at Yale and the Hospital of Rockefeller Institute, studying pneumococcus and respiratory illnesses. In 1930, Dr. Thomas Francis Jr. and William Tillett discovered C-reactive protein (CRP) in patients' blood, which interacts with pneumococcus carbohydrates to elicit a precipitation reaction [5,6]. This substance acts as a vital clinical marker, helping to assess infectious processes like rheumatic fever and providing critical insights into disease progression. Dubos and Avery's research on an induced enzyme from a soil bacterium, developed by Francis, reveals its potential in combating pneumococcus type III infections. The enzyme targets and breaks down capsular polysaccharides, highlighting their potential. Francis and Terrell developed techniques to induce type III pneumonia in primates, demonstrating its healing abilities in monkeys. However, researchers did not conduct a human trial to evaluate its therapeutic impact [7]. CRP is an acute-phase reactant produced by the liver in response to inflammation, often following an infection. Its ability to interact with the "C" carbohydrate found on the surface of pneumococcus triggered a precipitation reaction, providing an early indicator of infection and inflammation long before modern diagnostic tools were available.

The clinical significance of CRP was evident, as it served as a valuable marker for assessing the presence of acute infections and inflammatory conditions, from pneumonia to rheumatic fever and other systemic inflammatory diseases. Physicians could use CRP levels to monitor the intensity of the inflammatory response, providing a tool to gauge disease progression or evaluate the effectiveness of treatment.

Francis also demonstrated that the capsular polysaccharides of Streptococcus pneumoniae could induce protective antibodies in humans, a breakthrough for immunology. This concept of immunization laid the groundwork for the development of pneumococcal vaccines, which would later save countless lives, particularly in vulnerable populations such as older people, children, and those with weakened immune systems.

His work transcended individual discoveries, expanding our understanding of how the body responds to bacterial infections and how vaccines can prevent disease. His discovery of CRP and the immunogenic properties of pneumococcal polysaccharides advanced the science of infection and immunity and laid the foundation for modern clinical diagnostics and vaccine development. CRP remains an essential biomarker used in clinical practice to monitor infection, inflammation, and autoimmune diseases.

Thomas Francis Jr. may not have been the original creator of the first pneumococcal vaccine. Still, his groundbreaking work in immunology and vaccine development and his understanding of bacterial diseases like Streptococcus pneumoniae greatly influenced the area. The continued creation of vaccinations to prevent pneumococcal infections and other infectious illnesses is a testament to his work.

Work of Thomas Francis on Influenza Vaccine

Thomas Francis considered identifying the virus causing the influenza-like epidemic in Puerto Rico in 1934. He developed a method for obtaining sputum samples from individuals exhibiting symptoms similar to influenza, which resulted in the first effective isolation of the influenza virus in a lab environment. Francis and his colleague Dr. Thomas Magill demonstrated the antigenic diversity of influenza A viruses in 1936, showing that various strains may display unique surface antigens. This significant discovery in the knowledge of the flu virus established the foundation for the idea of antigenic drift and shift, which would later be essential in elucidating the necessity of yearly flu vaccinations. However, the scientific community viewed this notion with mistrust.

Another significant finding was made by Francis in 1940 when he was able to isolate influenza B, a virus that had previously been challenging to differentiate from influenza A. Increasing our knowledge of influenza and laying the foundation for the creation of vaccinations that might protect against both influenza A and B were made possible by this. Using tissue samples and animal models (such as mice), Francis also expertly cultivated influenza A and B viruses in lab conditions.

Since his appointment as Director of the Commission on Influenza at the Army Epidemiological Board (AFEB), he designed a polyvalent influenza vaccine that his team created that showed exceptional efficacy in protecting military troops against several strains of influenza A in 1943. In 1945, he also established vaccination against influenza B [2].

Through his contributions to the fundamental scientific understanding of the virus and his pivotal role in the practical application of this knowledge through the creation of vaccinations, Thomas significantly changed the battle against influenza. His work on vaccine development, antigenic diversity, and virus isolation has had a long-lasting effect on public health.

Role of Thomas Francis in the Development of Polio Vaccine

In 1950, Dr. Jonas Salk discovered the first inactivated polio vaccine [8]. This vaccine was used to kill Poliovirus and help to stimulate an immune response without causing disease. In 1952, during the worst outbreak in the United States, the influenza virus had infected 6,000 children and resulted in 3,000 deaths. On average, the illness incapacitated 45,000 individuals annually within the USA alone [9]. Thomas Francis was named head of the field trials to evaluate the effectiveness of Jonas Salk's polio vaccine in 1954. Approximately, 1.8 million children nationwide participated in these studies, which at the time were one of the biggest and most comprehensive public health programs [10-12]. Francis contributed to the development of the trial's procedures, which made it possible to precisely evaluate the vaccine's efficacy and safety. His leadership and careful preparation were crucial to the trial's success.

In April 1955, Francis was the one who revealed the experiment results, demonstrating the vaccine's effectiveness and safety. An important turning point in the fight against polio was reached when this announcement led to the widespread distribution of the vaccine. His declaration effectively marked the beginning of widespread polio vaccine efforts, which significantly reduced the prevalence of the disease both domestically and internationally.

As the principal investigator of the 1954 field tests that demonstrated the safety and efficacy of the Salk polio vaccine, Thomas Francis made a significant contribution. In many regions of the world, polio was almost completely eradicated thanks to his leadership and knowledge, which helped transform the vaccine's theoretical promise into a public health success. Many people believe that his work is essential to the worldwide struggle to fight the illness.

Achievement

In 1954, he was elected as a member of the American Philosophical Society. In 1946, he was honored with the USA Presidential Medal of Freedom [13]. In 1947, he was presented with the Lasker Award [14]. In 1948, he was selected for membership in the United States National Academy of Science (USNAS). In recognition of his work on polio research, he earned a nomination to the Polio Hall of Fame in 1958. In 1960, the American Academy of Arts and Sciences selected him for membership. In 1963, he joined the Jane Coffin Childs Memorial Fund for Medical Research's Board of Scientific Advisors, and from 1965 to 1969, he held the position of Director of the Board [15]. In 2005, the University of Michigan dedicated Thomas Francis Jr. by creating the Thomas Francis Jr. Medal in Global Public Health [16]. He was a famous epidemiologist and well-known for his work in the field of infectious diseases and vaccines. He published many papers available in databases like PubMed and Google Scholar.

## Conclusions

Dr. Francis was a famous physician, virologist, and epidemiologist. He was a trailblazer in the fields of infectious disease research and vaccine development. His research on polio, influenza, and pneumonia established the groundwork for contemporary immunology and public health procedures. The discovery of CRP by Francis was pivotal in diagnosing and tracking illnesses like pneumonia and rheumatic fever, as it served as an early sign of infection. His idea helped in the development of pneumococcal vaccines, which are still saving lives today, based on his studies of Streptococcus pneumoniae and its capsular polysaccharides. His work on influenza, which included isolating the influenza virus and proving antigenic variation in influenza A, broadened our knowledge of viral illnesses. His contribution to the isolation of influenza B also helped develop vaccines to prevent influenza A and B. Francis led the 1954 field with tests of the Jonas Salk polio vaccine, which proved safe and efficient. As a result, health authorities widely distributed the vaccine, and the incidence of polio drastically decreased globally. His research had a major impact on epidemiology and vaccine development, saving millions of lives and enhancing world health, making him a pioneer in vaccinology.
